# Lipidomics dataset of *Danio rerio* optic nerve regeneration model

**DOI:** 10.1016/j.dib.2021.107260

**Published:** 2021-07-14

**Authors:** Jennifer Arcuri, Matthew B. Veldman, Sanjoy K. Bhattacharya

**Affiliations:** a Bascom Palmer Eye Institute, Miller School of Medicine at University of Miami, Miami, FL, 33136, USA; b Miami Integrative Metabolomics Research Center, Miami, FL, 33136, USA; c Molecular Cellular Pharmacology Graduate Program, University of Miami, Miami, FL, 33136, USA; d Department of Cell Biology, Neurobiology, and Anatomy, Medical College of Wisconsin, Milwaukee, WI 53226, USA

**Keywords:** Regeneration, CNS Injury, Optic Nerve, Zebra-fish, Lipids

## Abstract

The right optic nerve of adult, 6 month to 1 year old, female and male *Danio rerio* were crushed and collected three days after. Matching controls of uninjured left optic nerves were also collected. The tissue was dissected from euthanized fish and frozen on dry ice. Samples were pooled for each category (female crush, female control, male crush, male control) n = 24 to obtain sufficient tissue for analysis. The brain from one male fish was also collected for control/calibration. Lipid extraction was done with the Bligh and Dyer [1] method, followed by untargeted liquid chromatography-mass spectrometry (LC MS-MS) lipid profiling using a Q-Exactive Orbitrap instrument coupled with Vanquish Horizon Binary UHPLC LC-MS system. The lipids were identified and quantified with LipidSearch 4.2.21 and the statistical analysis was conducted through Metaboanalyst 5.0. This data is available at Metabolomics Workbench, Study ID ST001725.

## Specifications Table

**Table:** 

Subject	Ophthalmology
Specific subject area	Lipids of the regenerating optic nerve
Type of data	Table Image Chart Graph Figure Chromatograms Spectra
How data were acquired	Liquid Chromatography Q-Exactive Orbitrap Mass Spectrometry
Data format	Raw Analysed Filtered
Parameters for data collection	Lipid profiling was performed on 3 day post crush injury and uninjured control optic nerves from adult, 6 month to 1 year old, zebra-fish of both genders.
Description of data collection	The right optic nerve was crushed in anesthetized, 6 month to 1 year old zebra-fish. The fish recovered from anesthesia in fresh water and were placed back into the aquarium system for 3 days. On the third day tissue was dissected from euthanized fish and frozen on dry ice in Eppendorf tubes. The optic nerves from 24 fish were pooled, for each category (Crush Female, Crush Male, Uninjured Female, Uninjured Male).
Data source location	Bascom Palmer Eye Institute, Miller School of Medicine at University of Miami, Miami, FL 33136, USA
Data accessibility	Study ID ST001725 at Metabolomics Workbench Repository https://www.metabolomicsworkbench.org/data/DRCCMetadata.php?Mode=Study&StudyID=ST001725 MetaboAnalyst 5.0 (https://www.metaboanalyst.ca/) web based free tool that can be used to analyse these data.

## Value of the Data

• The data provided shows the lipid changes that occur in the optic nerve of zebrafish during regeneration after crush compared to uninjured nerves.
• The data is useful for understanding the lipidomic changes associated with successful optic nerve regeneration.
• Damage of the central nervous system in humans tends to result in permanent loss of function while Adult zebra-fish regenerate and regain functionality. This dataset provides insights into the regulatory changes that could be used for therapeutic development.

## 1 Data Description

**Fig. 1:**
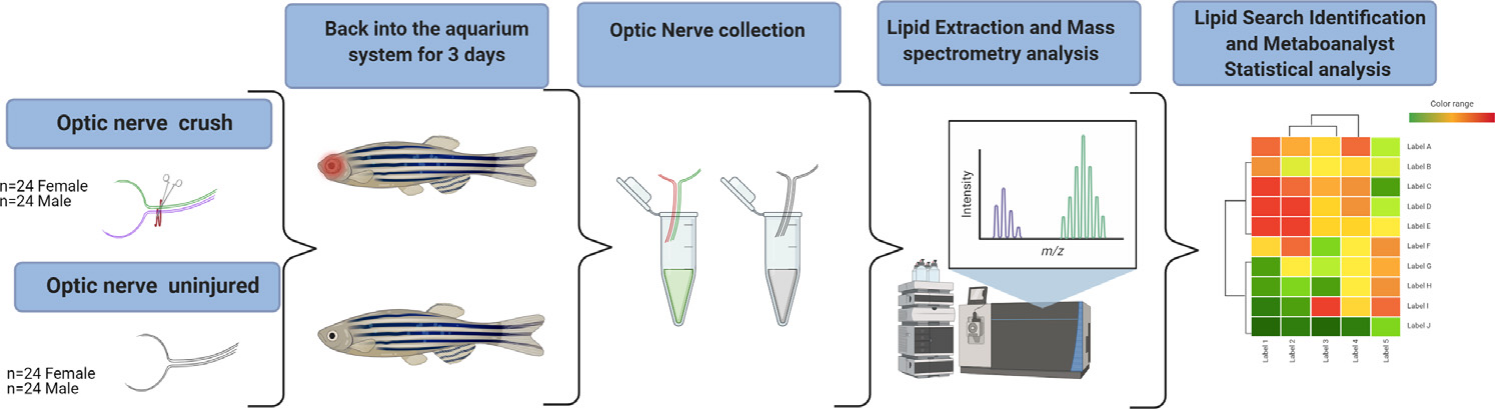
Schematic representation of the lipid profiling protocol followed for optic nerve regeneration in zebrafish.

**Fig. 2:**
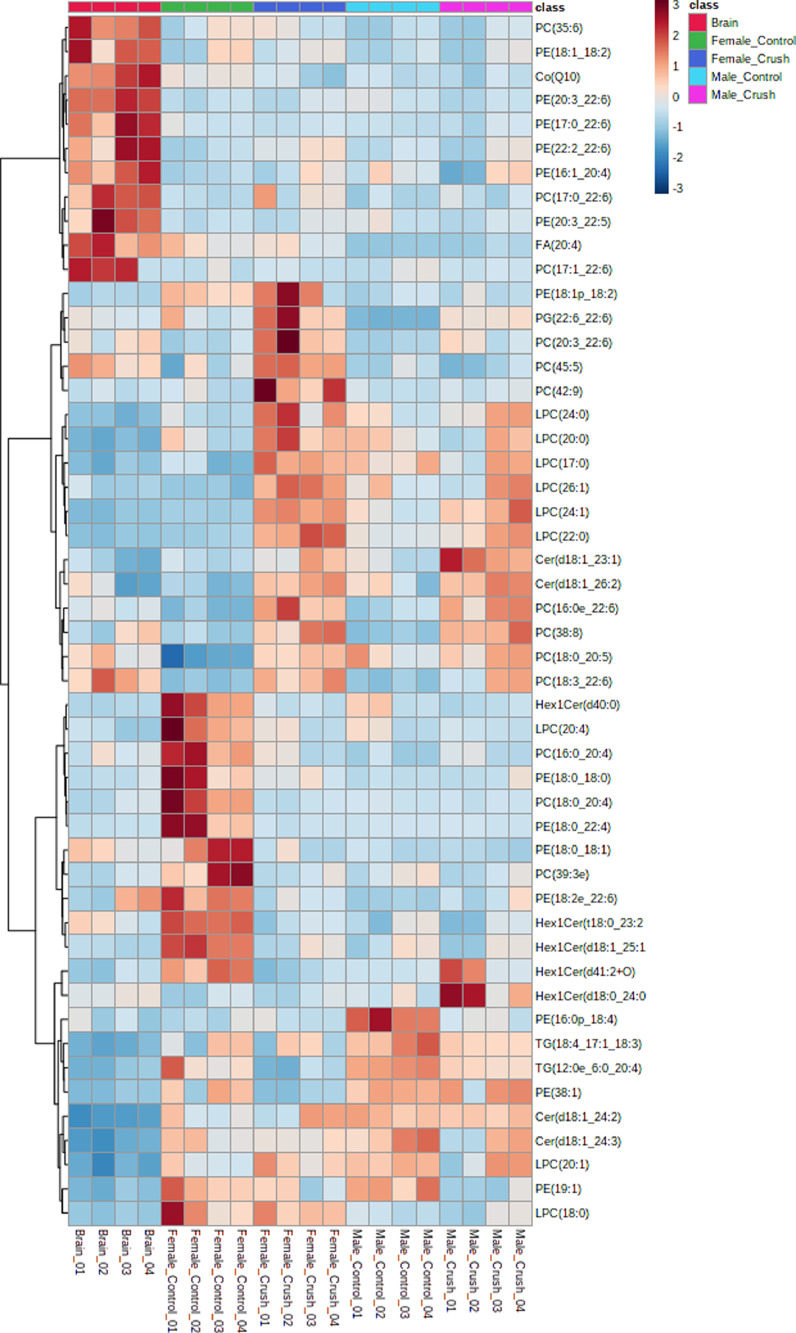
Heatmap of the lipid concentration changes in the optic nerve of zebrafish in both genders, with and without crush. A male brain was also included as control.

**Fig. 3:**
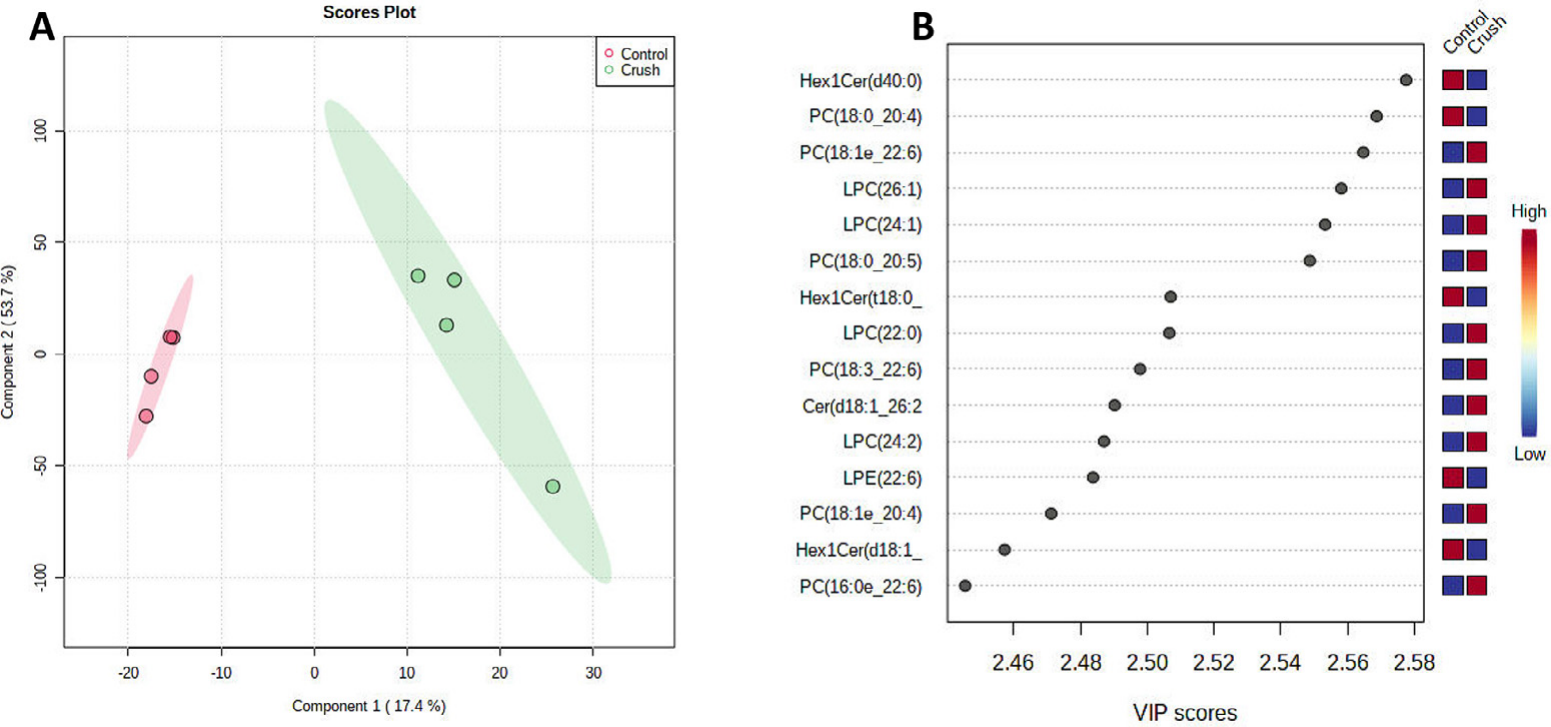
The statistical features of lipid profiles of control vs. crush zebra fish optic nerves. Important features identified by partial least square-discriminant analysis (PLS-DA) and variable importance in projection (VIP) scores have been presented (A-B). The colored boxes on the right indicate the relative concentrations (Red is high and Blue is low) of the lipid species in control vs. crush.

**Fig. 4:**
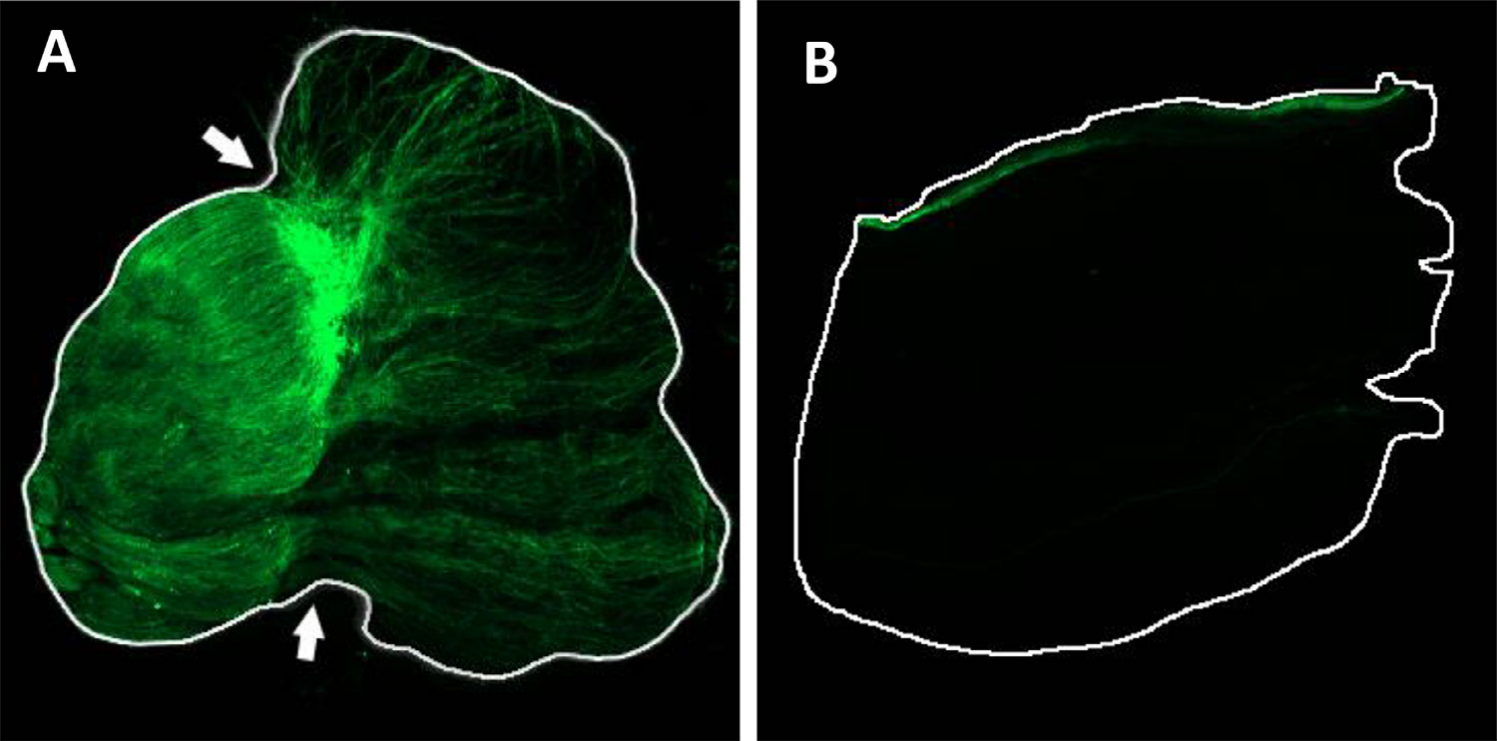
Optic Nerve Regeneration Visualization on *Tg(gap43:GFP)* transgenic zebrafish, 3 days post crush (A) and control (B).

Lipid profiling was performed on 3 day post crush injury and uninjured control optic nerves from adult, 6 month to 1 year old, zebra-fish. The samples were divided by gender and due to the small size, 24 optic nerves were pooled per category. The brain from one male fish was also collected for control/calibration. Lipid extraction was done with the Bligh and Dyer [2] method. From the extracted lipids, four technical replicates were made to be used for mass spectrometry. Untargeted liquid chromatography-mass spectrometry (LC MS-MS) lipid profiling was performed using a Q-Exactive Orbitrap instrument coupled with Vanquish Horizon Binary UHPLC LC-MS system. Each sample replicate was run in positive and negative ion mode. The lipids were identified and quantified with LipidSearch 4.2.21 and deposited on Metabolomics Workbench (Study ID ST001725). The identified lipids were exported and provided in the supplementary table. For further details on the lipid names abbreviation, please refer to our previous paper [1]. The statistical analysis was conducted through Metaboanalyst 5.0 (Fig. 1). The heatmap of all samples done by distance measure using Euclidean, and clustering algorithm using ward.D shows the top 50 species (Fig. 2). Further analysis of the crush vs. control regardless of gender by important features identified by partial least square-discriminant analysis revealed the top 15 lipid species (Fig. 3). Visualization of the expected regeneration was carried on *Tg(gap43:GFP)* [3] transgenic zebrafish (Fig. 4).

## 2 Experimental Design, Materials and Methods

### 2.1 Animals

Adult zebra-fish, 6 months to 1 year in age, were used for all groups both male and female. The NIH guide for the care and use of Laboratory animals was followed and all procedures were approved by the Medical College of Wisconsin IACUC ID AUA1378. For optic nerve crush, animals were deeply anesthetized in 0.033% tricaine methane-sulfonate (MS-222). The right optic nerve was exposed by gently removing the connective tissue on the dorsal half of the eye and rotating the eye ventrally out of the orbit with a number 5 forceps. A nerve crush was then performed using number 5 forceps to crush the nerve ~0.5–1 mm from the optic nerve head for 5 s. Success was observed by the generation of a translucent stripe in the nerve that completely separated two areas of white myelination with no bleeding. Fish were then revived in fresh aquatic system water in individual tanks. After 1 h the tanks were returned to the fish system and animals were maintained normally with daily feeding until 3 days post injury. For tissue collection, animals were euthanized by overdose of MS-222 and optic nerve removed by dissection from the optic nerve head to the optic chiasm.

### 2.2 Optic Nerve Regeneration Visualization

To demonstrate the expected amount of axonal regeneration present in the samples, optic nerve crush was performed on *Tg(gap43:GFP)* [3] transgenic zebra-fish. On day 3 post crush injury the animal was euthanized, and the optic nerves dissected out. After fixation in 4% PFA for 1 h at room temperature, the nerves were flat mounted on a slide and imaged on a NikonC2 confocal microscope for GFP fluorescence.

### 2.3 Lipid Extraction

Lipids were isolated from optic nerves with the Bligh and Dyer [2] method. The lower organic phase containing the lipids was removed and dried in a Speed-Vac (Model 7810014; Labconco, Kansas City, MO). To prevent lipid oxidation, the tubes were flushed with argon gas for storage.

### 2.4 High Performance Liquid Chromatography and Mass Spectrometry

The dried samples containing the lipids were stored in −80 °C until re-suspension in 50 µl of chloroform: methanol 1:1 (v/v), followed by a 20 min ultrasonic water bath. For Mass Spectrometry analysis, 24 µl were taken from each sample and placed in two glass vials, one with 2 ug/ml of EquiSPLASH™ LIPIDOMIX® Quantitative Internal Standard (330731). The Vanquish Horizon UHPLC system (Thermo) was used for reversed phase chromatographic separation with an Accucore Vanquish C18+ column. The column and sample tray temperature were held at 55 °C and 4 °C. The Q Exactive (Thermo) mass spectrometer was operated under heated electrospray ionization (HESI) and samples were ran in both positive and negative mode separately. The solvents were LC-MS grade methanol: water 60:40 (v/v) with 10 mM ammonium acetate and methanol chloroform 60:40 (v/v) with 10mM ammonium acetate.

### 2.5 Lipid Identification and Statistical Analysis

The lipids were identified and quantified from the *.RAW scans using Lipid Search 4.2.21 software (Thermo). The parameters were set to M-score 2 and the predefined product search module for Q-Exactive. The quantification was carried for each lipid species, according to the class from the deuterium labeled lipid internal standard used. In Metab-analyst 5.0, the auto-scale feature was employed (mean-centered and divided by standard deviation of each variable).

## Ethics Statement

Studies in humans and animals. This study utilized animals (Zebrafish, encompassing both genders) only. All animal experiments were performed in compliance with the US National Institutes of Health guide for the care and use of Laboratory animals. The sex of the fish is not known to influence or have an association with optic nerve neuron regeneration.

## CRediT Author Statement

**Jennifer Arcuri:** Experimentation, Data curation, Writing – original draft preparation; **Matthew Veldman:** Animal experimentation, Writing – reviewing & editing; **Sanjoy Bhattacharya:** Conceptualization, Methodology, Software, Writing – reviewing & editing.

## Declaration of Competing Interest

The authors declare that they have no known competing financial interests or personal relationships which have, or could be perceived to have, influenced the work reported in this article.

## Appendix Supplementary materials

Image, application 1
